# (Fear of) SARS-CoV-2 infection and psychological distress: a population-based cohort study in southern Switzerland

**DOI:** 10.1017/S2045796024000507

**Published:** 2024-11-28

**Authors:** C. Sculco, B. Bano, G. Piumatti, R. Amati, C. Barbui, L. Crivelli, M. Purgato, E. Albanese

**Affiliations:** 1 Institute of Public Health, Università della Svizzera Italiana, Lugano, Switzerland; 2 WHO Collaborating Centre for Research and Training in Mental Health and Service Evaluation, Department of Neurosciences, Biomedicine and Movement Sciences, University of Verona, Verona, Italy; 3 Fondazione Agnelli, Turin, Italy; 4 Department of Business Economics, Health and Social Care, University of Applied Sciences and Arts of Southern Switzerland, Lugano, Switzerland

**Keywords:** common mental disorders, epidemiology, mental health, psychological assessment, stressful life events

## Abstract

**Aims:**

It is widely recognized that the COVID-19 pandemic exerted an impact on the mental health of the general population, but epidemiological evidence is surprisingly sparse. We aimed to explore the association between serologically confirmed SARS-CoV-2 infection and psychological distress – assessed by symptoms of depression, anxiety and stress – in the general adult population in southern Switzerland, a region widely affected by the pandemic. We also investigated whether this association varied over time and between pandemic waves from late 2020 through 2021.

**Methods:**

We used data from 305 adults who participated in the Corona Immunitas Ticino prospective seroprevalence study in southern Switzerland, including results of the serological tests of SARS-CoV-2 infection collected in June 2021, and explored associations with depression, anxiety and stress scores as measured by the 21-item Depression, Anxiety and Stress Scale at three time points between December 2020 and August 2021, accounting for socio-demographic and health characteristics.

**Results:**

In our sample, 84.3% of the participants (mean age of 51.30 years, SD = ±.93) were seronegative at baseline. Seropositive (i.e., infected) participants had a decreasing probability of being depressed and anxious through the COVID-19 pandemic waves compared to the seronegative (non-infected) participants. Further, seropositivity at baseline was also associated with more rapid decline in depressive, anxiety and stress symptomatology, and younger age and the presence of chronic diseases were independently associated with mild anxiety (OR = .97; *P* = 0.013; 95% CI = 0.95, 0.99; OR = 3.47; *P* = 0.001; 95% CI = 1.71, 7.04) and stress (OR = .96; *P* = 0.003; 95% CI = .94, .99; OR = 2.56; *P* = 0.010; 95% CI = 1.25, 5.22).

**Conclusions:**

Our results suggest that the MH consequences of the pandemic may not be due to the SARS-CoV-2 infection per se, but to fears associated with the risk of infection, and to the pandemic uncertainties.

## Introduction

SARS-CoV-2 infection has been associated with a variety of psychological symptoms including low mood, anxiety and stress (Witteveen *et al.*, 2023), to which the COVID-19 pandemic itself has also greatly contributed through prolonged and forced isolation and strict rules determining fear, and social isolation and deprivation (Hornstein and Eisenberger, 2022). Hundreds of millions worldwide have been infected, and billions have been affected by the pandemic. Moreover, because these effects may have endured (Thompson *et al.*, 2022) the impact of the pandemic on the population’s mental health (MH) remains a major public health reason of concern.

An ample and wide-ranging corpus of evidence showed that depressive symptoms, anxiety and psychological distress were common during the early stages and through the COVID-19 pandemic in the general population (Thompson *et al.*, 2022; Witteveen *et al.*, 2023). Still, evidence on the long-term psychological consequences of the COVID-19 pandemic is somewhat patchy and scanty (Blankenburg *et al.*, 2022; Jafri *et al.*, 2022; Larsen *et al.*, 2022; Natarajan *et al.*, 2023; Osaghae *et al.*, 2021). Postolache *et al.*, (2021) suggested mechanistic underpinnings include the neurotropism of the SARS-CoV-2 virus, which would cause direct detrimental effects on the Central Nervous System (CNS). Neurological symptoms of COVID-19 and SARS-CoV-2 infection were common in infected individuals, including dysgeusia and ageusia (i.e., gustatory, and olfactory dysfunctions), myalgia, headache, confusion, delirium and dizziness (Harapan and Yoo, 2021), and may relate to psychological distress (including symptoms of depression, anxiety and stress) to the body’s neuroendocrine and immune systems (Peters *et al.*, 2021; Steenblock *et al.*, 2020) and consequent wane of the immune response (Segerstrom and Miller, 2004). Somewhat surprisingly, epidemiological evidence on the prospective association between infection (i.e., non-vaccine-induced seropositivity to SARS-CoV-2 antibodies) and psychological distress remains extremely sparse.

Leveraging epidemiological data of a population-based cohort, the aim of this study was to explore the prospective association between SARS-CoV-2 infection and facets of psychological distress (i.e., anxiety, depressive and stress symptomatology) among non-institutionalized adults aged 20 years and older, across the pandemic waves. Our hypothesis is that SARS-CoV-2 infection leads to enduring mental distress, which is lower in non-infected compared to infected individuals.

## Methods

### Study design and participants

This study stems out of Corona Immunitas Ticino (CIT), a prospective population-based seroprevalence study conducted in southern Switzerland during the COVID-19 pandemic. The CTT study was previously described (Amati *et al.*, 2022) and is part of Corona Immunitas (Speierer *et al.*, 2022; West *et al.*, 2020), a national research program conducted to assess the population-level spread and impact of the COVID-19 pandemic in Switzerland. For this study, we focused on a representative sample, randomly drawn by the Swiss Federal Statistical Office, of adults (aged 20–64 years) and older individuals (65+ years) living in Ticino (southern Switzerland), with socio-demographic baseline data collected in July 2020, serological data on immune status collected in June 2021 and repeated psychological distress assessments (21-item Depression, Anxiety and Stress Scale [DASS-21], below) collected from December 2020 up until August 2021, that is from the first through the second and third pandemic waves in the region. All participants gave written informed consent to participate in the study.

### Measurements and procedures

At study entry, we collected information on the socio-demographic and health characteristics of participants, including age, categorized into three age groups: (0) 20–49, (1) 50–64, (2) 65+ years; gender: (0) women, (1) men; education: (0) up to higher secondary/apprenticeship, (1) higher tertiary; body mass index (BMI): (0) BMI < 30 kg/m^2^, (1) BMI > 30 kg/m^2^; smoking status: (0) nonsmoker/former smoker, (1) current smoker (daily or occasional); and existing chronic conditions (‘Do you suffer from any of the following chronic conditions?’): (0) none, (1) any among hypertension, diabetes, cardiovascular disease, cancer, immunological deficiency syndromes or respiratory syndromes.

Serological testing of the CTT studies is described in detail elsewhere (Amati *et al.*, 2023), and all assays were previously validated in population-based samples (Fenwick *et al.*, 2021b, 2021b). Briefly, we obtained sera from peripheral venous blood samples and conducted longitudinal serosurveys at five time points between July 2020 and June 2022. In this study, we used data from the third serosurvey conducted in June 2021, when the vaccination campaign in the region was already ongoing. We assessed SARS-CoV-2 specific antibodies against the spike and nucleocapsid proteins of the virus using Sensitive Anti-SARS-CoV-2 Spike Trimer Immunoglobulin Serological (SenASTrIS), a Luminex binding assay for anti-SARS-CoV-2 total immunoglobulins, purposely developed for population-based serosurveys (Amati *et al.*, 2023; Fenwick, Croxatto, *et al.*, 2021; Fenwick, Turelli, *et al.*, 2021). This assay allowed the distinction between infection and/or vaccine-induced immunity. The assay measures binding of IgG antibodies to the trimeric SARS-CoV-2 spike and the nucleocapsid proteins. The test has a high specificity (98%) and sensitivity (99%) and has been validated in samples of the general population and in specific subgroups (Fenwick *et al.*, 2021b, 2021b).

Based on the serological results we classified immunological statuses as follows: (i) Infection-induced immunity (self-reported vaccination status = NO, Anti_Spike = POS and/or Anti_N = POS); (ii); Vaccine-induced immunity (self-reported vaccination status = YES, and/or Anti_Spike = POS and Anti_N = NEG); (iii) Hybrid immunity (self-reported vaccination status = YES, Anti_Spike = POS and Anti_N = POS); (iv) Seronegative (Anti_Spike = NEG e Anti_N = NEG, irrespective of vaccination status). For this study, we were interested in infections and further dichotomized the samples in never infected individuals (seronegative individuals and individuals with vaccine-induced immunity only) and infected individuals (infection-induced individuals and individuals with hybrid immunity).

For the outcome, and dependent variable in our models, we considered three complete assessments of psychological distress using the DASS-21 for depressive symptoms, anxiety and stress levels (Henry and Crawford, 2005) over the different seasons of the COVID-19 pandemic: winter (December 2020–February 2021); spring (March–May 2021) and summer (June–August 2021). Each DASS-21 item is self-rated on a 4-level Likert scale, from 0 (never) to 3 (almost always). The DASS-21 was used in previous research on psychological distress associated with COVID-19 (Piumatti *et al.*, 2022; Wang *et al.*, 2021). We computed the DASS-21 overall score, which ranges between 0 and 21, and we used standard cutoffs of the three subscales’ scores for mild levels of depressive symptoms (>9), anxiety (>7) and stress (>14) (Lovibond and Lovibond, 1995; Tran *et al.*, 2013) and obtained dichotomized measures of each score accordingly. We modelled the assessments of DASS-21 as a dichotomized score (Piumatti *et al.*, 2022) (normal and mild levels of anxiety, depression and stress vs moderate, severe to extremely severe levels of anxiety, depression and stress), we analyzed the outcome of each subscale separate (Lovibond and Lovibond, 1995; Tran *et al.*, 2013), and we used mild conditions as a standard reference cut-off score for assessing the occurrence of anxiety, depression and stress (i.e., mild [0] vs not mild levels [1]). The DASS-21 has good convergent, discriminant and nomological validity in normative samples (Henry and Crawford, 2005; J. Lee *et al.*, 2019); Cronbach’s alpha ranged from 0.89 to 0.93 for depression, from 0.76 to 0.86 for anxiety and from 0.89 to 0.93 for stress across assessments.

We collected and recorded all data using secured online questionnaires and forms implemented in the Research Electronic Data Capture (REDCap) software, hosted at the Università della Svizzera Italiana (USI) (2019; Harris *et al.*, 2009).

### Statistical analysis

We checked data quality (i.e., straight line scoring) and analysed missing data patterns (Piumatti *et al.*, 2022). We excluded responses due to straight line scoring on the DASS-21 items (<0.4% across assessments), and participants with incomplete assessment of DASS-21 i.e., less than three assessments over different seasons as mentioned above (*n* = 773, 37.5.5% of the study sample), and derived an analytic sample of 305 individuals in which we conducted all analysis. Next, we imputed missing values of repeated measures of depression, anxiety and stress using linear combination of available observations (Piumatti *et al.*, 2022). Additionally, we compared the infected and never infected characteristics with chi2 and mean Students’t-tests, as appropriate.

We modelled moderate to severe depression, anxiety and stress as binary dependent variables in separate generalized estimating equation (GEE) models to assess variance structure and clustering error within subjects (Liang and Zeger, 1986; Ziegler and Vens, 2010). GEE models allow the determination of how the average of a subject’s response changes with covariates while specifying variance structure for the correlation between repeated measurements in the same subject over time (Piumatti *et al.*, 2022). To select the best working covariance structure for the current data, we followed a model selection method described elsewhere (Cui, 2007; Pan, 2001): smaller quasi-likelihood under the quasi-information criterion values was indicative of better fit. We assessed three types of covariance structure (Grady and Helms, 1995): exchangeable, assuming responses from the same cluster are equally correlated; autoregressive, where correlations between responses decrease across time; and unstructured, considering the correlations between responses to be comparatively complex. We tested GEE univariate models with robust standard errors adjusted for age and gender. We then adjusted also for education, BMI, smoking and chronic diseases in multivariate models (Piumatti *et al.*, 2022). We further tested significant between-subject effects in interaction with time and plotted results to ease interpretation. Statistical significance was considered for *P* < 0.05 for direct effects and *P* < 0.10 for interaction effects. We used Stata version 15, for all statistical analyses (StataCorp., 2015).

## Results

**Figure 1.**
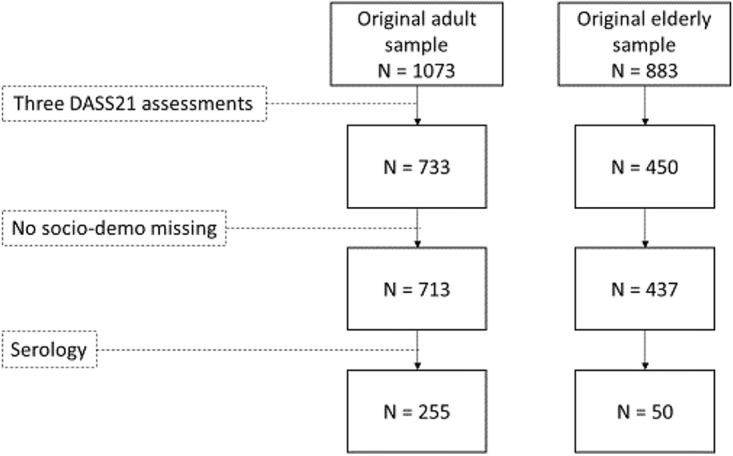
Participants’ flow chart.

We included only participants with at least three DASS-21 assessments (*n* = 733 adults; *n* = 450 older adults) and we excluded those with incomplete socio-demographic information (*n* = 20 adults; *n* = 13 older adults) and with no serological data in June 2021 (*n* = 458 adults; *n* = 387 older adults). We obtained a final sample of 305 participants (see Fig. 1 – flow chart).

### Description of the sample

Table 1 reports the characteristics of the analytical sample by immunological status. From the total sample (*n* = 305) of infected and never infected (as dichotomized in the ‘Measurements and procedures’ section), 84.3% were never infected and had a mean age of 51.3 years (SD = 0.93). The infected participants were slightly younger on average (M = 46.9; SD = 2.0). 50.2% of the never infected and 45.8% of the infected were female, and in both groups the minority of the participants had a tertiary level of education or higher (28.40% and 31.25%, respectively). And 15.2% of the never infected and 18.8% of the infected participants were obese (BMI > 30 kg/m^2^), 24.5% and 22.9% reported a previous clinical diagnosis of at least one chronic disease and 17.5% of the never infected and 14.6% infected individuals were current smokers. The two groups did not significantly differ in any baseline socio-demographic characteristics other than age distribution (*χ*^2^ = 6.229; *P* = 0.04).

**Table 1.** Characteristics of the analytical sample at baseline (July 2020) by infection status, Corona Immunitas Ticino (CIT) study

Sample characteristics	Never infected (%)	Infected (%)	*N* (*n*)	*χ*^2^	*P* value*
**Total**	84	16	305		
**Age groups, years**				6.23	0.04*
20–49	42	50	131		
50–64	40	46	124		
65+	19	4	50		
**Gender**				0.31	0.58
Female	50	46	151		
Male	50	54	154		
**Education**				0.16	0.69
High education	28	31	88		
Low education	72	69	217		
**Obese (yes)**	15	19	48	0.39	0.53
**Chronic (yes)**	25	23	74	0.06	0.81
**Smoking (yes)**	18	15	52	0.24	0.62

* *P* < 0.05 (Pearson *χ*^2^) as significant.

### SARS-CoV-2 infection and psychological distress

**Figure 2.**
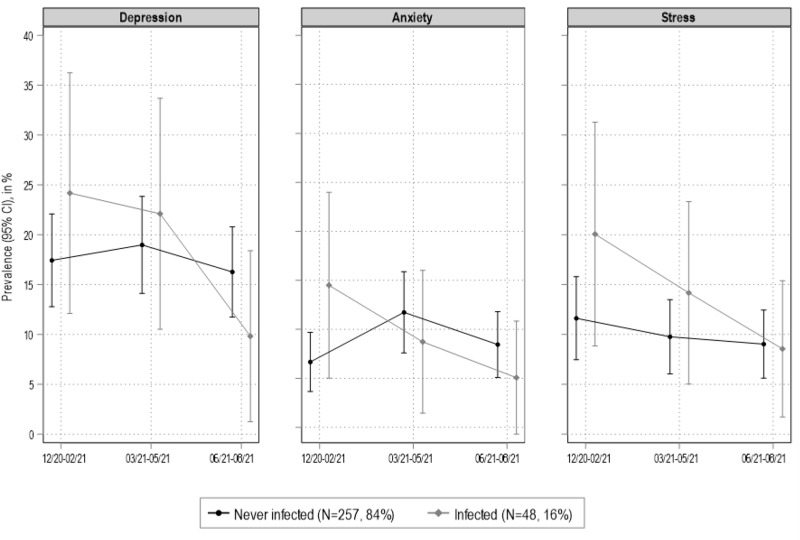
Generalized estimating equation (GEE) models results (DASS-21 cut-offs for mild conditions). Covariates are age, gender, education, BMI, smoking and chronic diseases. Ticino, southern Switzerland (*N* = 305).

*Note*: Generalized estimating equation (GEE) models results. Covariates include time, age, gender, chronic diseases, BMI, smoking and education level.

Table 2 reports the GEE regression results with an exchangeable variance–covariance structure, which fitted the data better than an autoregressive or unstructured solution to model the effect of infection on change in psychological distress (Piumatti *et al.*, 2022). Compared with those who were never infected, infected individuals had a decreasing probability of being mildly depressed (DASS-21 sub-score >9) ([OR] = 0.64; 95% CI = 0.45, 0.91; *P* = 0.014) and anxious (OR = 0.50; 95% CI = 0.27, 0.94; *P* = 0.030) (DASS-21 sub-score > 7) through the COVID-19 pandemic waves. On the contrary, infected individuals did not show a declining probability of reporting mild stress symptoms (OR = 0.71; 95% CI = 0.47, 1.08; *P* = 0.113) (DASS-21 sub-score > 14). Trends over time (Fig. 2) of depressive symptoms, anxiety and stress declined faster in infected compared to never infected individuals.

**Table 2.** Associations (odds ratios) between seropositive immunological status and mental health between December 2020 and August 2021 in Ticino, southern Switzerland (*N* = 305)

DASS-21 defined mild condition	OR	*P*-value	95% CI
Depression	0.641	0.014	0.449–0.914
Anxiety	0.502	0.030	0.270–0.936
Stress	0.712	0.113	0.468–1.083

Figure 2 provides a graphical illustration of the putative effect of infection on trajectories of psychological distress, based on the GEE models results, which are presented as prevalence (95% CI) of mild depression, anxiety and stress based on the DASS-21 sub-scales with standard cut-offs for mild-conditions (depression > 9; anxiety > 75; stress > 14). None of the associations of infection status with depressive symptoms, anxiety and stress levels reached statistical significance when modelled separately at the three follow-ups (all *P*-values of independent, unadjusted regression models >0.051). Trends across time were in favor of infected individuals indeed, when examining the figure, it appeared that psychological distress decreased more rapidly in infected compared to never infected individuals. Similarly, in summer 2021 (i.e., 6 months after the first MH assessment) scores of depressive symptoms, anxiety and stress levels declined faster in infected compared to never infected individuals.

In mutually adjusted GEE models, younger age (OR = 0.97; 95% CI = 0.95, 0.99; *P* = 0.013), self-reported chronic diseases (OR = 3.47; 95% CI = 1.71, 7.04; *P* = 0.001) and smoking (OR = 2.52; 95% CI = 1.23, 5.16; *P* = 0.011) were all significantly associated with higher levels of anxiety. Similarly, younger age (OR = 0.96; 95% CI = 0.94, 0.99; *P* = 0.003) and self-reported chronic conditions (OR = 2.56; 95% CI = 1.25, 5.22; *P* = 0.010) were significantly associated with increased stress scores. None of the associations between socio-demographic characteristics and depressive symptoms reached statistical significances (all *P*-values >0.140).

## Discussion

The aim of this study was to explore the prospective association between SARS-CoV-2 infection and depression, anxiety and stress symptomatology. Our results may suggest a psychological pathway linking infection to distress. However, we found that, compared to never infected individuals those who were infected had a progressive improvement in psychological distress symptoms (i.e., depressive symptoms, anxiety and stress) from December 2020 to late August 2021. Moreover, younger age (age ranges: 20–49; 50–64; 65+), presence of chronic diseases and smoking habits were all independently associated with anxiety and stress symptoms over time.

Previous evidence about the impact of the COVID-19 infection on long-term MH symptoms is heterogenous but limited on the prospective association of serologically confirmed infection with psychological distress. Most studies focused on MH features in SARS-CoV-2 cases compared to non-cases (Jafri *et al.*, 2022; Magnúsdóttir *et al.*, 2022; Ray *et al.*, 2021) not on infectious status. Moreover, although previous studies focused on diverse populations including both clinical (Ray *et al.*, 2021) and community-dwelling samples (Blankenburg *et al.*, 2022; Fogh *et al.*, 2022; Magnúsdóttir *et al.*, 2022), many were conducted in healthcare workers or settings (Grazzini *et al.*, 2022; Osaghae *et al.*, 2021). Study designs also varied, ranging from cross-sectional (Jafri *et al.*, 2022; Larsen *et al.*, 2022), case-control (Burrai *et al.*, 2021) to cohort (Blankenburg *et al.*, 2022; Magnúsdóttir *et al.*, 2022; Osaghae *et al.*, 2021). Varying study designs, populations and time periods likely explain the marked heterogeneity of findings across studies, that reported positive (Jafri *et al.*, 2022; Thompson *et al.*, 2022) and null (Blankenburg *et al.*, 2022; Larsen *et al.*, 2022; Osaghae *et al.*, 2021) associations between SARS-CoV-2 infection and psychological distress. Our findings are based on longer observational periods compared to those of a cohort study (Osaghae *et al.*, 2021) in which serology was tested during spring 2020 and MH outcomes (i.e., depression and anxiety) in summer 2020 (i.e., after 6 and 16 weeks from baseline), and which similarly did not identify any significant difference in MH outcomes between seronegative and seropositive adults. Similar results were obtained in studies (Blankenburg *et al.*, 2022; Larsen *et al.*, 2022) that employed other MH measures including the Patient Health Questionnaire-9 and General Anxiety Disorder-7 questionnaire (Osaghae *et al.*, 2021), or investigated different population groups, such as the adolescents (Blankenburg *et al.*, 2022).

Our findings that psychological distress was higher in infected compared to never infected individuals only in winter 2020–2021 might suggest a short-term, direct effect of the virus on MH and may be due to its neurotropism, but also that COVID-19 infection may cause psychological distress because of the uncertainty in the ensuing disease course and/or fear of infecting others. Nevertheless, psychological distress decreased more rapidly in infected compared to never infected individuals. This contradicts, in part, our hypothesis. It is possible that the latter feared future infections, and that this fear of infection contributed to sustained psychological distress. This is consistent with consolidated evidence on the putative causative role of fear in depression, anxiety and stress (Folayan *et al.*, 2022). Fear of COVID-19 infection, specifically, may underpin and cause anxiety, depressive and stress symptoms (Alimoradi *et al.*, 2022; Bakioğlu *et al.*, 2021; Luo *et al.*, 2021). Because the impact of COVID-19 was greater on older than younger adults (Thompson *et al.*, 2022), fear of infection would plausibly increase with age too. Therefore, our observations on the inverse association between age and psychological distress were somewhat unexpected and may be explained, at least to some extent, by better coping attitudes in older compared to younger adults (Derrer-Merk *et al.*, 2023).

Fear of infection and uncertainty on the disease course may explain why infected individuals were initially stressed and progressively, as uncertainty about the disease course diminished, less distressed compared to never infected individuals. To this end, our findings would not support current hypothesis on the short- and long-term effects of the virus. Further investigations are warranted because the neurological symptoms and consequences of the SARS-CoV-2 are well known (Brola and Wilski, 2022; Harapan and Yoo, 2021; Hosseini *et al.*, 2021) and they include among others, anosmia, ischaemic and haemorrhagic stroke, headache, hypoxia and meningitis. That psychological distress was not associated with infection but was pervasive in our study sample, endured several months through the pandemic waves and was explained to some extent by socio-demographic and health characteristics is worth noting and may have considerable public health implications because it suggests a prominent role of the pandemic itself, conceived as collective traumatic events of unprecedented proportions. Worldwide, billions of lives were overturned by the pandemic, significantly more than the hundreds of millions who were ultimately infected.

Our study has limitations. First, our sample was not homogenous in terms of serostatus, the never infected outnumbered the infected. However, this reflects the actual infection spreading in 2021. Second, serology and MH symptomatology were assessed concomitantly only for the third DASS-21 measurement; hence, we cannot exclude that the serology status of some participants may have changed during the study. Similarly, we cannot uncover any individual who potentially got infected but whose antibodies were not detected (false negative; asymptomatic). Further, we were unable to identify how long ago the ‘infected participants’ exactly had the infection aside from when we retrieved the serology status. Consequently, the generalizability of our results is limited by the specificity of the study sample and the constraints associated with the assessments conducted (i.e., serology status and DASS-21). Though robust, and valid, the DASS-21 scale is not a diagnostic instrument; other facets of psychological distress might have been better captured with a structured diagnostic instrument such as the Minnesota Multiphasic Personality Inventory (Butcher, 2010). Still, we intentionally focused on symptoms of common mental disorders in the general population, not on psychopathology and/or diagnosis. Third, selection bias cannot be excluded. Our results may underestimate or overestimate the true prevalence of moderate to severe depression, anxiety and stress in the target population because severe psychological distress and psychopathology could reduce individuals’ participation and increase attrition in studies involving frequent, though self-reported assessments. Compared to individuals without mood-related symptoms, those with more severe psychological distress may have been less likely to participate and more likely to drop out. This risk is outlined in the Cochrane collaboration Risk of Bias II assessment (Higgins *et al.*, 2019). Fourth, our results may be generalized only to populations with MH profiles and infection status like those of our sample. Even tough, group differences in our sample were not statistically significant for any socio-demographic characteristics other than age, further research will be needed to assess the relationship between BMI and gender differences as potential confounding variables. Fifth, we did not use a formal assessment of fear or specific scales like, for example, the Corona Virus Anxiety Scale (Lee, 2020). Our interpretation of the role of fear is speculative. Yet, the DASS-21 provides good proxies of fear-related distress. Further, the study did not consider any distinction among SARS-CoV-2 variants. However, we assessed seropositivity in mid-2021, when infections were mainly due to gamma and delta variants (Ufficio federale della anità pubblica, 2023). Finally, we did not differentiate between acute or overcome infections as it was beyond the scope of this study.

Strengths of our study include the use of a validated and comprehensive tool for measuring MH outcomes. The DASS-21 offers a comprehensive evaluation of the three main dimensions associated with psychological distress (i.e., depression, anxiety, stress), which represent the most common psychological symptoms reported during the pandemic (Alqahtani *et al.*, 2023). Further, we performed repeated assessments in a population-based (non-clinical) sample, which was representative of the general population. Compared to studies that focused on clinical samples (Ray *et al.*, 2021) or specific populations, i.e., adolescents (Blankenburg *et al.*, 2022) and healthcare workers and/or settings (Grazzini *et al.*, 2022; Osaghae *et al.*, 2021), our results may be less biased and have greater external validity. We applied robust statistical techniques to capture temporal variations and longitudinal patterns of psychological distress over one year of observation during the COVID-19 pandemic, and formally confirmed the goodness of fit of our statistical models. Therefore, that our initial hypothesis was not confirmed seems unlikely due to type 2 error (i.e., missing true association when present), also because we did find significant inverse associations between infection and psychological distress symptoms.

## Conclusions

The public health implications of our study relate to the importance of lessening the overall impact of potential future pandemic (or similar) events conceived as a collective traumatic experience. Considering the limitations inherent in our study sample, our observations suggest that long-term MH consequences of the pandemic may not be due to the SARS-CoV-2 infection, but plausibly to the uncertainties and fears associated with the risk of infection. Our findings are novel, and replications are warranted, but our study highlights the importance of MH preventive components within preparedness strategies for potential future pandemics or other public health emergencies.

## Supporting information

Sculco et al. supplementary material Sculco et al. supplementary material

## Data Availability

Data will be available upon reasonable request through a methodologically sound proposal directed to the corresponding author (Emiliano Albanese, emiliano.albanese@usi.ch).

## Supplementary material

The supplementary material for this article can be found at https://doi.org/10.1017/S2045796024000507.
